# The Adaptor Protein Grb2 Is Not Essential for the Establishment of the Glomerular Filtration Barrier

**DOI:** 10.1371/journal.pone.0050996

**Published:** 2012-11-30

**Authors:** Nicolas Bisson, Julie Ruston, Marie Jeansson, Rachel Vanderlaan, W. Rod Hardy, Jianmei Du, Samer M. Hussein, Richard J. Coward, Susan E. Quaggin, Tony Pawson

**Affiliations:** 1 Samuel Lunenfeld Research Institute, Mount Sinai Hospital, Joseph and Wolf Lebovic Health Complex, Toronto, Ontario, Canada; 2 Department of Molecular Genetics, University of Toronto, Toronto, Ontario, Canada; 3 Division of Nephrology, St. Michael’s Hospital, and Department of Medicine, University Health Network, University of Toronto, Toronto, Ontario, Canada; INSERM, France

## Abstract

The kidney filtration barrier is formed by the combination of endothelial cells, basement membrane and epithelial cells called podocytes. These specialized actin-rich cells form long and dynamic protrusions, the foot processes, which surround glomerular capillaries and are connected by specialized intercellular junctions, the slit diaphragms. Failure to maintain the filtration barrier leads to massive proteinuria and nephrosis. A number of proteins reside in the slit diaphragm, notably the transmembrane proteins Nephrin and Neph1, which are both able to act as tyrosine phosphorylated scaffolds that recruit cytoplasmic effectors to initiate downstream signaling. While association between tyrosine-phosphorylated Neph1 and the SH2/SH3 adaptor Grb2 was shown *in vitro* to be sufficient to induce actin polymerization, *in vivo* evidence supporting this finding is still lacking. To test this hypothesis, we generated two independent mouse lines bearing a podocyte-specific constitutive inactivation of the *Grb2* locus. Surprisingly, we show that mice lacking Grb2 in podocytes display normal renal ultra-structure and function, thus demonstrating that Grb2 is not required for the establishment of the glomerular filtration barrier *in vivo*. Moreover, our data indicate that Grb2 is not required to restore podocyte function following kidney injury. Therefore, although *in vitro* experiments suggested that Grb2 is important for the regulation of actin dynamics, our data clearly shows that its function is not essential in podocytes *in vivo*, thus suggesting that Grb2 rather plays a secondary role in this process.

## Introduction

Kidney glomeruli are composed of a network of anastomosing capillaries, which constitute an important filtration barrier to allow the passage of water and solutes while retaining plasma macromolecules. This glomerular wall is formed by three layers: endothelial cells, basement membrane (GBM), and specialized epithelial cells, podocytes [1]. Podocytes extend long, actin-based protrusions, called foot processes that interdigitate to cover the glomerular capillaries [2]. As a result, podocyte foot processes form a specialized tight junction, the slit diaphragm, which is an important part of the filtration barrier.

A number of proteins reside in the slit diaphragm, notably the transmembrane proteins Nephrin and Neph1 [3]. Nephrin is encoded by *NPHS1*, the gene mutated in Congenital Nephrotic Syndrome of the Finnish variety, a disorder characterized by massive proteinuria and nephrosis [4]. Nephrin was shown to serve as a tyrosine phosphorylated scaffold that recruits proteins to the cytoplasmic face of the foot processes. Src-family kinases, in particular Fyn, were shown to phosphorylate several tyrosine residues on the cytoplasmic tail of Nephrin [5], [6], thus creating docking sites for a number of Src-homology 2 (SH2) domain-containing proteins such as Crk [7], Nck [8], phosphatidyl-inositol-3-kinase (Pi3k) regulatory subunit [9] and phospholipase C (Plc)-γ [10].

The simplest function of SH2-SH3 adaptor proteins such as Crk, Grb2 and Nck is to mediate protein-protein interactions. Through their SH3 domains, these proteins are able to bind effectors harboring poly-proline motifs, and link them to phosphorylated tyrosine (pTyr)-containing proteins, to which adaptors are bound via their single SH2 domain. Our group has shown that adaptors Nck1/2 are essential to connect phosphorylated Nephrin with the actin cytoskeleton in podocyte foot processes, and their function is required during both podocyte development and maintenance [8], [11], [12]. More recently, it was reported that podocytes lacking Crk1/2 develop normally, but display an increased resistance to foot process effacement in a model of induced nephropathy [8].

Neph1 was also found to be tyrosine phosphorylated in podocytes and to regulate actin cytoskeleton dynamics. While not able to bind Nck1/2, Neph1 was shown to directly associate with Grb2, the best characterized of the SH2/SH3 adaptor proteins [11], [13], [14], [15]. Grb2 was originally described as linking activated Receptor Tyrosine Kinases (RTKs) to Ras-MAPK signaling via the membrane recruitment of the Sos guanine nucleotide exchange factor [16]. The physiological importance of mammalian Grb2 in mediating RTK signaling was demonstrated by its involvement in the differentiation of primitive endoderm in mice, which requires functional SH2 and SH3 domains; mice lacking Grb2 do not survive past E3.5 [17]. In cultured podocytes, Grb2 was shown to form functional complexes with Sos and the CIN85 scaffold [18].

The association between Tyr-phosphorylated Neph1, but not Nephrin, and Grb2 was shown to be sufficient to induce actin polymerization in cells. It was also proposed that the Neph1-Grb2 pair cooperates with Nephrin-Nck to transmit more efficiently signals leading to actin polymerization [11]. Therefore, this evidence suggested Grb2 function to be essential in regulating actin dynamics during podocyte development or establishment of a response to kidney injury. We have tested this hypothesis *in vivo* by generating mice bearing a podocyte-specific constitutive inactivation of the *Grb2* locus. We report that mice lacking Grb2 in podocytes show no signs of proteinuria or nephrosis and display a normal renal ultra-structure. Our results demonstrate that Grb2 is not required for the establishment of the glomerular filtration barrier *in vivo*, and suggest that Grb2 function is neither essential for phospho-tyrosine signaling nor for regulating the actin cytoskeleton in podocytes.

## Results

### Grb2 is Expressed in Mouse podocytes *in vivo*

**Figure 1:**
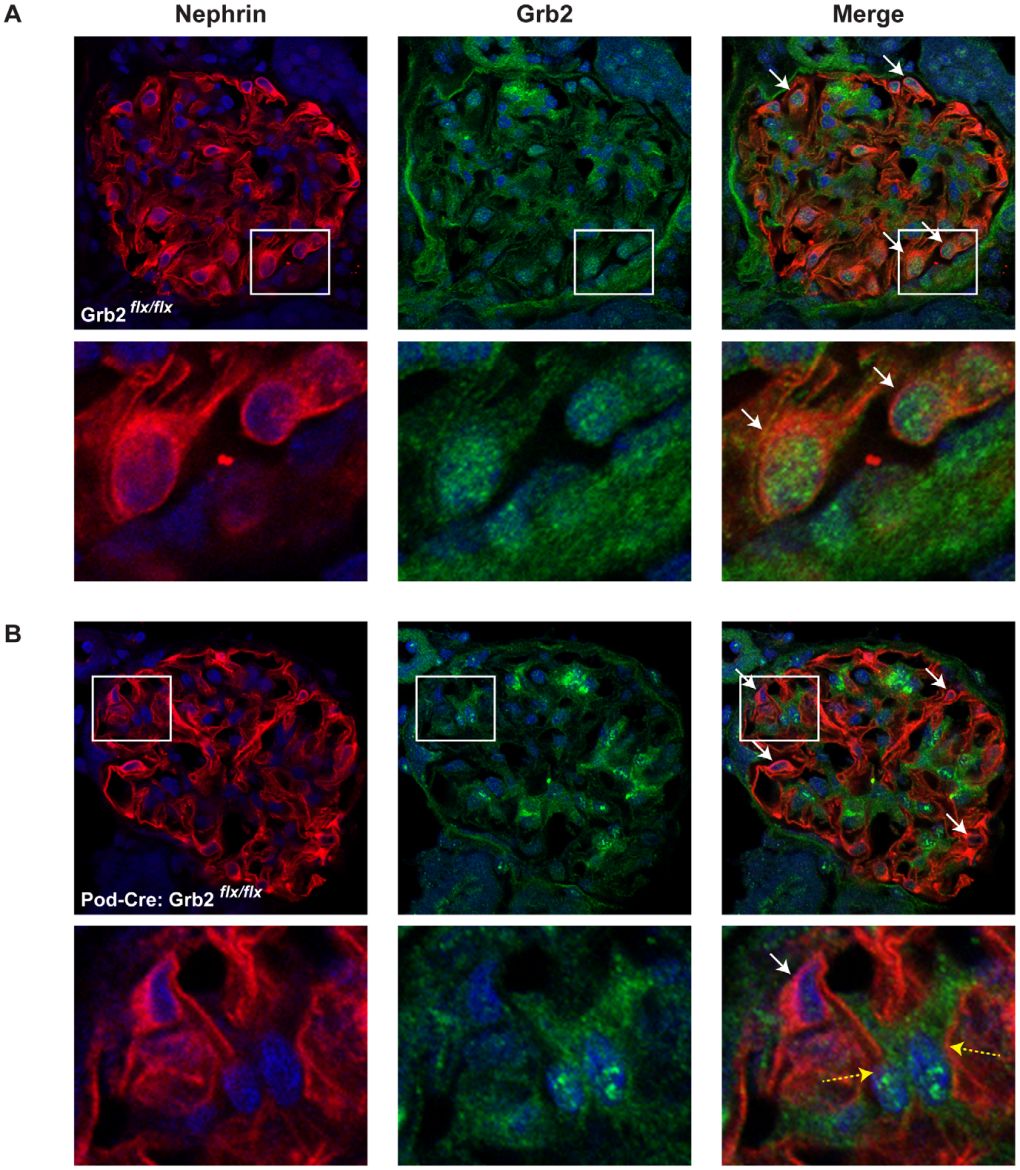
Grb2 is expressed in wild-type mouse podocytes. Immunofluorescent staining for Nephrin and Grb2 in a wild-type (A, *Grb2^flx/flx^*) or mutant (B, *Podocin*-Cre;*Grb2^flx/flx^*) glomerulus. Arrows indicate nephrin-expressing podocyte cells located at the periphery of the glomerular tuft. Framed areas from top panels are shown at a higher magnification below. Grb2 staining is absent from *Podocin*-Cre;*Grb2^flx/flx^* podocytes. Dashed yellow arrows in B show nephrin-negative non-podocyte cells that retained Grb2 expression.

We first analyzed by immunofluorescence the distribution of Grb2 in whole mouse glomeruli. We found that Grb2 has a pronounced expression in both cytoplasmic and nuclear compartments of podocytes, which are located at the periphery of the glomerular tuft (Figure 1A). We also observed that Grb2 is expressed in the presumptive mesangial compartment, located at the center of the tuft.

### Generation of Podocyte-specific *Grb2* knock-out Mice

**Figure 2:**
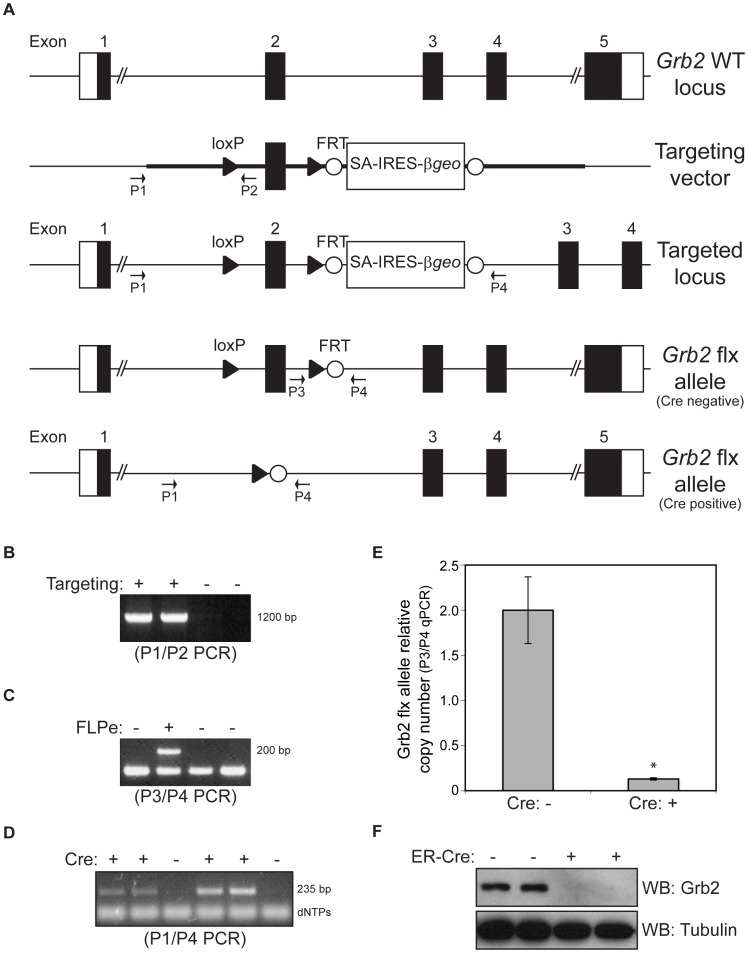
Conditional inactivation of the *Grb2* gene in mice. (A) Schematic representation of the *Grb2* locus targeting strategy and the resulting conditional *Grb2^flx^* allele. LoxP sites are represented with white triangles and FRT sites with white circles. Genotyping primers P1 to P4 are shown. (B) Example of 2 positive ES clones targeted at the *Grb2* locus, as judged from a positive P1/P2 PCR product of 1200 bp. (C) Example of a successful FLPe-mediated excision of the SA-IRES-β*geo*-pA cassette to generate the *Grb2^flx^* allele, as judged from a positive P3/P4 PCR product of 200 bp. (D) PCR analysis of Cre-mediated excision of the *Grb2^flx^* allele in mouse glomeruli. A 235 bp P1/P4 PCR product confirms excision at the locus and correlates with the presence of Cre recombinase. (E) qPCR analysis of Cre-mediated excision of the *Grb2^flx^* allele in FACS-sorted podocytes from Podocin-Cre; Nephrin-CFP; Grb2flx/flx (mutant, Cre+, n = 2) or Nephrin-CFP; Grb2flx/flx (control, Cre-, n = 3) mice. Amplification levels of a P3/P4 PCR product were normalized to B-actin and used to calculate relative copy numbers of the non-excised *Grb2* flx allele (Cre-: 2.00±0.37 and Cre+: 0.13±0.01). Star represents p-value of 3.8E-05. (F) Western blot showing 2 examples of *ROSA*-CreER-mediated inactivation of the *Grb2^flx^* allele in MEFs, resulting in the absence of the *Grb2* protein product. Positive Cre (+) indicates treatment with OHT to activate the expression of the transgene.

In order to analyze the involvement of Grb2 in podocyte development, we first generated mice bearing a podocyte-specific constitutive inactivation of the *Grb2* locus (Figure 2A–C). *Podocin*-Cre^+^; *Grb2* ^flx/flx^ animals were viable and obtained in accordance to Mendelian inheritance ratios (Table 1). A PCR approach on isolated glomerular gDNA suggests that the *Grb2* allele is excised in Cre-expressing animals (Figure 2D). To quantify the excision events, we introduced into our mice a Nephrin-CFP transgene that we used to enrich, by FACS, podocytes from enzymatically dissociated glomeruli. Using qPCR on gDNA from the FACS-sorted CFP+ podocytes, we measured the relative copy numbers of the non-excised *Grb2* floxed allele in *Podocin*-Cre; *Nephrin*-CFP; *Grb2* ^flx/flx^ animals compared to *Nephrin*-CFP; *Grb2* ^flx/flx^ (Cre-) controls. We calculated that only 0.13±0.01 copy (out of 2) of the *Grb2* flx allele remain non-excised (Figure 2E). This corresponds to an average excision of 93.4%. In addition, we derived fibroblasts from Grb2^flx/flx^ mouse embryos (MEFs), in which we ectopically expressed a hydroxy-tamoxifen (OHT)-inducible CreER recombinase. Treatment of cells with OHT resulted in a complete loss of Grb2 protein after 72 hours, thus confirming that the floxed allele of Grb2 is correctly and completely excised by Cre recombinase (Figure 2F). Podocyte-specific gene inactivation of *Grb2* was further confirmed by immunofluorescence. We did not detect Grb2 in either the cytoplasmic or nuclear compartments of podocytes in *Podocin*-Cre; *Grb2* ^flx/flx^ animals (Figure 1B).

**Table 1:** Genotype analysis of the progeny born from *Podocin*/*Nephrin*-Cre;*Grb2^flx^* crosses.

Podocin-Cre+;Grb2^flx/wt^ X Grb2^flx/flx^				
Genotype	Cre+;flx/flx	Cre+;flx/wt	Cre−;flx/flx	Cre−;flx/wt
Observed frequency (%)	21.8 (n = 39)	20.6 (n = 37)	25.1 (n = 45)	32.4 (n = 58)
Expected frequency (%)	25	25	25	25
**Nephrin-Cre+;Grb2^flx/wt^ X Grb2^flx/flx^**				
**Genotype**	**Cre+;flx/flx**	**Cre+;flx/wt**	**Cre−;flx/flx**	**Cre−;flx/wt**
Observed frequency (%)	33.0 (n = 43)	26.9 (n = 35)	19.2 (n = 25)	20.8 (n = 27)
Expected frequency (%)	25	25	25	25

### Loss of Grb2 does not Impair Podocyte Development and Kidney Function

**Figure 3:**
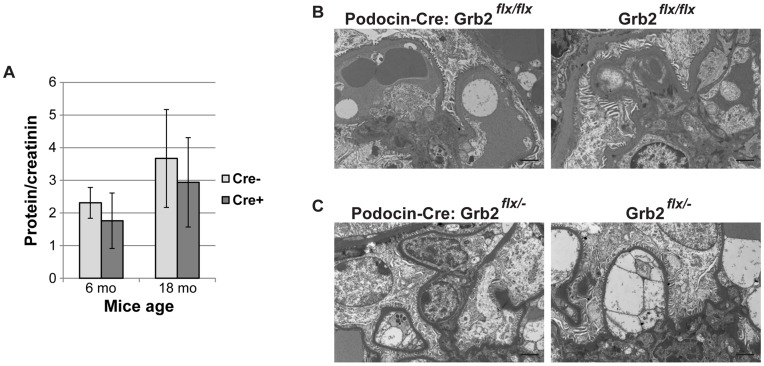
*Podocin*-Cre;*Grb2^flx/flx^* animals display normal glomerular structure and renal function. (A) Urinalysis of *Podocin*-Cre;*Grb2^flx/flx^* (Cre+) and *Grb2^flx/flx^* (Cre-) mice 6 and 18 months after birth. Average measurements and standard deviations were calculated for 3–5 mice from each group. (B–C) Representative examples of ultra-structural electron microscopy analysis of kidneys of *Podocin*-Cre; *Grb2^flx/flx^* (A) and *Podocin*-Cre: *Grb2^flx/−^* (B) animals compared to control (Cre-negative) littermates. Bar is 2 µm.

Unexpectedly, *Podocin*-Cre^+^; *Grb2* ^flx/flx^ mice did not show signs of proteinuria, even 18 months after birth (Figure 3A). Renal histology of these animals was similar to Cre-negative controls (data not shown). Furthermore, ultra-structural studies by electron microscopy (EM) did not reveal alterations in glomerular organization as podocytes were clearly surrounding capillaries, with no signs of effacement (Figure 3B). *Podocin*-Cre^+^; *Grb2* ^flx/−^ animals showed the same wild-type (WT) phenotype (Figure 3C).

**Figure 4:**
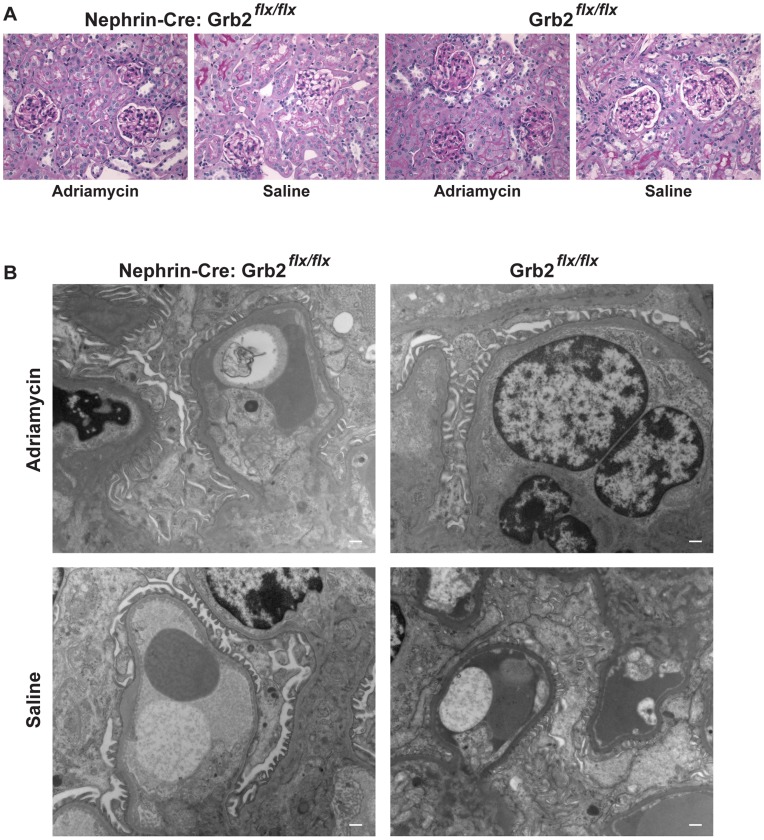
Loss of Grb2 does not impair podocyte development in *Nephrin*-Cre; *Grb2^flx/flx^* animals. (A) *Nephrin*-Cre; *Grb2^flx/flx^* animals, injected with adriamycin or saline, display a normal renal histology 9 weeks after treatment, as visualized by periodic acid–Schiff (PAS)-stained paraffin kidney sections. (B) Representative examples of ultra-structural electron microscopy analysis of kidneys of *Nephrin*-Cre; *Grb2^flx/flx^* animals, injected with adriamycin or saline, compared to control (Cre-negative) littermates, 9 weeks after treatment. Bar is 500 nm.

To further confirm our observations, we generated *Grb2* ^flx/flx^ mice expressing a Cre recombinase under the control of a different podocyte-specific promoter, from the *Nphs1* gene (*Nephrin*). As observed for *Podocin*-Cre^+^ animals, *Nephrin*/Cre^+^; *Grb2* ^flx/flx^ mice were viable and the genotypes obtained from the progeny were as expected for Mendelian inheritance (Table 1). None of our *Grb2* ^flx/flx^ Cre-positive animals displayed signs of kidney disease, as judged from the absence of proteinuria, no weight loss (Table S1), normal renal histology and no glomerular ultra-structure abnormalities (Figure 4). These observations confirmed our findings with *Podocin*-Cre^+^; *Grb2* ^flx/flx^ animals that Grb2 is not required for the establishment of the glomerular filtration barrier *in vivo*.

### Grb2 is not Required for the Recovery of the Kidney Following Injury

In order to determine if Grb2 function is required in the response to renal injury, a process for which actin cytoskeleton reorganization is crucial, we injected *Grb2* ^flx/flx^ mice with adriamycin, a chemical that induces nephropathy [19]. This led to massive proteinuria and/or body weight loss within 5 days, in most animals (10/14) of either Cre-positive or negative genotypes (Table S1), compared to saline-injected controls. Five adriamycin-treated animals died within a few weeks following severe weight loss, without correlation of genotypes. Analysis of renal histology and glomerular ultra-structure of the remaining mice 9 weeks post-injection did not reveal significant defects (Figure 4). We detected a slight increase in sclerosis and matrix expansion; however, we did not observe significant differences between *Grb2^flx/flx^* Cre-positive and Cre-negative mice (data not shown). In agreement with a functional intact filtration barrier, electron microscopy (EM) showed that Grb2^flx/flx^ Cre-positive mice have intact podocyte foot processes with slit diaphragms. A single adriamycin-treated Grb2^flx/flx^ Cre-positive mouse displayed swollen endothelium and increased GBM thickness in one capillary loop. Therefore, we conclude that Grb2 is not required to restore podocyte function following kidney injury.

## Discussion

We have presented evidence that the adaptor protein Grb2 function is not required during podocyte development. Podocyte-specific excision of the *Grb2* locus was performed independently in two mouse lines, with Cre recombinase expression driven by promoters commonly utilized, i.e. *Nephrin* (*NPHS1*) and *Podocin* (*NPHS2*). In both mouse lines, Grb2 inactivation did not lead to proteinuria and did not induce any alteration in glomerular function and structure. Therefore, while *in vitro* experiments suggested that Grb2 is important for the regulation of actin dynamics in podocytes, our data unexpectedly but clearly show that its function is not essential *in vivo*.

In the mouse genome, two Grb2-related adaptor proteins, namely Grap1 (Grap) and Grap2 (Gads), are present and may have compensated for the inactivation of the Grb2 locus, thus providing an explanation for the lack of phenotype. However, the expression of both Grap1 [20] and Grap2 [21] was shown to be restricted to hematopoietic cells (thymus and spleen). It is also clear from our previous work [17] that the two proteins cannot compensate for the loss of Grb2 in early embryos. Although it is very unlikely that in our podocyte model, Grap1 and Grap2 are able to compensate for the loss of Grb2, this possibility may not be excluded without further experiments.

The function of another SH2-SH3 adaptor protein, Nck, is required downstream of Nephrin to establish and maintain the glomerular filtration barrier in mouse [8], [12]. When compared to Nephrin-Nck, evidence pointing towards a lesser importance for the Neph1-Grb2 pair in podocyte function, in addition to the data presented here, is twofold. First, although gene inactivation of *Neph1* in mouse suggests its importance for podocyte function [22], no mutation was found in patients to date. While Grb2 represents the leading candidate to convey signals downstream of Neph1, it also remains a possibility that it is not Grb2 but another protein that is responsible for bridging Neph1 to the actin cytoskeleton. A bioinformatics analysis of the protein sequence of the cytoplasmic tail of Neph1 using Scansite [23] revealed the presence of proline-based motifs that may act as putative docking sites for one of the SH3 domains of the adaptor/scaffolds Crk and Plc-γ. These proteins have previously been reported to associate with a Tyr-phosphorylated form of Nephrin [7], [10], [14], and to interact with Wave1 and N-Wasp actin nucleating proteins, respectively [24], [25]. Hence, they represent possible candidates linking Neph1 with the actin cytoskeleton, in a pTyr-independent manner.

Second, Grb2 associates with Neph1 via a single pTyr, and N-Wasp with Grb2 possibly via two SH3 domains. As a comparison, 3 pTyr sites present on Nephrin may each be a potential docking site for Nck SH2 domain, and each of the 3 SH3 domains of Nck is able to bind N-Wasp. Interestingly, it was reported that the multiple pTyr Nck-SH2 binding sites on Nephrin and the multiple SH3 domains of Nck act cooperatively to create multivalent complexes that promote actin polymerization in cells [26]. This important function of the Nephrin-Nck pair is also consistent with mutations on Nephrin found in patients with Congenital Nephrotic Syndrome, a phenotype that is recapitulated in Nck-deficient mice [8]. In contrast, we argue that the Neph1-Grb2 pair may not be leading to multivalent interactions, which are required for efficient actin nucleation by Nephrin-Nck complexes [26]. Consistent with this, it was suggested that clustered Neph1 does not lead to sufficient recruitment of N-Wasp to properly induce actin polymerization [11]. This is also supported by observations made using a different model, vaccinia virus. In this system, the virus attaches to its target cell and inserts the A36R protein (the so-called vaccinia actin tail nucleator), leading to the recruitment of Nck and Grb2 to Tyr112 and Tyr132, respectively. However, while Nck function is required to nucleate actin tails [27], Grb2 was rather found to play a secondary stabilizing role, being itself unable to induce actin reorganization in the host cell during virus infection [28], [29]. We propose that a similar mechanism operates in podocytes, wherein the absence of Grb2 may not lead to actin dynamics deficiencies that are severe enough to result in a loss of podocyte function and deficits of the kidney filtration barrier.

## Materials and Methods

### Mice

Mice with a *Grb2* allele (designated *Grb2^flx^*) that can be conditionally inactivated via Cre/loxP-mediated DNA recombination were generated by introducing into ES cells loxP sites flanking exon 2 of the *Grb2* gene using a SA-IRES-β*geo*-pA targeting cassette (Figure 1A). Correctly targeted ES clones were identified by PCR (Figure 1B). Flpe-mediated deletion of the drug selection cassette from the mouse germ line was accomplished by crossing mice containing the SA-IRES-β*geo*-pA cassette with CAGGS-FLPe delete mice. In resulting *Grb2^flx^* mice, Cre/loxP-mediated excision of exon 2 introduces a deletion/frameshift mutation that prematurely terminates Grb2 translation upstream of the essential SH2 domain, thereby generating a functionally-null allele. Mice were housed and manipulated according to the guidelines of the Canadian Council on Animal Care and experiments were approved by the Toronto Centre for Phenogenomics Animal Care Committee (AUP #0011a-H).

### Genotyping

Genotypes of animals were determined by PCR amplification of genomic DNA. For the Grb2 flx locus, primers lxP1 (5′-gaactgaatatgaatagtaagtcagactctgg) and lxP2 (5′- ggtgctggggcagaatccagggctttgtgcatgc) were used. The primer pair amplified a fragment of 140 bp from the WT allele and 237 bp from the targeted allele. For the Grb2 null allele, primers KO_A (5′-ttgggtccaggtgaacaccagga) and KO_B (5′-ccttctatcgccttcttgacgag) were used to amplify a fragment of 900 bp on the targeted allele. Primers Cre_A (5′-gttataagcaatccccagaaatg) and Cre_B (5′-ggcagtaaaaactatccagcaa) were used to detect the presence of the Cre transgene. Primers Grb2_P1 (5′-gcttgtgatcacagcacttgggagatgg) and Grb2_P4 (5′-ggtgctggggcagaatccagggctttgtgcatgc) were used to amplify a fragment of 235 bp produced by Cre-mediated deletion of exon 2. Primers CFP_A (5′-aagttcatctgcaccaccg) and CFP_B (5′- tccttgaagaagatggtgcg) were used to detect the presence of the CFP transgene.

### Cell Culture and Protein Preparations

Primary mouse embryo fibroblasts (MEFs) from *ROSA*-CreER; *Grb2* ^flx/flx^ E13.5 embryos were prepared as described [30]. Cells at the second passage were treated with 1 uM 4-hydroxy-tamoxifen (OHT) for 24 hours, and collected a further 48 hours later to prepare protein extracts using ice-cold lysis buffer as described previously [30].

### Glomerular Isolation and Podocyte Sorting

Kidneys were dissected from mice and kept in ice cold PBS. Glomeruli were isolated by pressing the tissue through a 106 µm sieve followed by collection of glomeruli on a 71 µm sieve. CFP-positive podocytes were dissociated and enriched by FACS as previously described [31].

### Urine Analysis

Urine protein/creatinine ratios were calculated by measuring protein (Bradford Assay, Biorad) and creatinine (Jaffe method, R&D Systems) in spot urine.

### Tissue Analysis

For histopathology, kidneys were dissected from mice and fixed for at least 24 hours in 10% formalin in PBS. Samples were progressively dehydrated and embedded in paraffin. Cross sections of 5 to 20 microns were cut and periodic acid–Schiff stained. For electron microscopy (EM), kidneys were dissected from mice, chopped into 1 mm cubes and fixed in 1.5% glutaraldehyde in 2% glutaraldehyde in 0.1 M sodium cacodylate buffer, rinsed in buffer, post-fixed in 1% osmium tetroxide in buffer, dehydrated in a graded ethanol series followed by propylene oxide, and embedded in Quetol-Spurr resin. Sections 100 nm thick were cut on an RMC MT6000 ultra-microtome, stained with uranyl acetate and lead citrate and viewed in an FEI Tecnai 20 TEM. For immunofluorescence, kidneys were snap-frozen in Tissue-Tek OCT compound (Sakura) and cryosectioned. Primary antibodies goat anti-nephrin (R&D Systems) and rabbit anti-Grb2 C-23 (Santa Cruz) were used as recommended by the manufacturer. Cryosections were counterstained with wheat germ agglutinin-Texas Red or concanavalin A-Alexa Fluor 647 conjugate (Life Technologies). Immunofluorescence was observed by confocal microscopy on a Nikon Eclipse Ti instrument.

### Adriamycin-induced Proteinuria

Adriamycin (15 mg/kg) or control saline was injected in tail vein of animals at day 0. Body weight was measured every 2 days for the first week, starting at day 1 post-injection, and weekly for 9 weeks. Histological analyses were performed at 9 weeks. Spot urine was collected concomitantly for analysis, as described above.

### qPCR Analysis

Genomic DNA was prepared from isolated fractions using Trizol according to the manufacturer’s instructions (Bio-Rad). Purified gDNA was amplified using REPLI-g kit (Qiagen). Each sample was run on a CFX384 Real Time System (Bio-Rad) at 4 different dilutions in triplicates for Grb2 excision and reference gene beta-Actin using LuminoCt® SYBR® Green qPCR ReadyMix (Sigma-Aldrich), and samples were prepared using a Janus automated liquid handling robot (Perkin-Elmer). Standard curves were calculated using genomic DNA from CFP negative cells post FACS-sorting. Primers were as follows: Grb2_P3 (5′- gaactgaatatgaatagtaagtcagactctgg), Grb2_P4 (5′- ggtgctggggcagaatccagggctttgtgcatgc), beta-Actin_A (5′-agtacgatgagtccggcccct), beta-Actin_B (5′-aaagggtgtaaaacgcagctcagt). The final Grb2 excision (P3/P4 primers) value was normalized to beta-Actin.

## Supporting Information

Table S1 Detailed body weight and protein/creatinin measurements of *Nephrin*-Cre; *Grb2^flx/flx^* animals, injected with adriamycin or saline, over a period of 9 weeks.
(XLSX)
